# Recent advances in nanoparticle-based targeting tactics for antibacterial photodynamic therapy

**DOI:** 10.1007/s43630-022-00194-3

**Published:** 2022-04-06

**Authors:** Brydie A. Thomas-Moore, Carla Arnau del Valle, Robert A. Field, María J. Marín

**Affiliations:** 1 School of Chemistry, University of East Anglia, Norwich Research Park, Norwich, NR4 7TJ UK; 2 Norwich Research Park Innovation Centre, Iceni Glycoscience Ltd, Colney Lane, Norwich, NR4 7GJ UK; 3 Department of Chemistry and Manchester Institute of Biotechnology, The University of Manchester, 131 Princess Street, Manchester, M1 7DN UK

**Keywords:** Antibacterial photodynamic therapy, aPDT, Bacteria, Targeting, Nanoparticles

## Abstract

**Abstract:**

The rise of antibacterial drug resistance means treatment options are becoming increasingly limited. We must find ways to tackle these hard-to-treat drug-resistant and biofilm infections. With the lack of new antibacterial drugs (such as antibiotics) reaching the clinics, research has switched focus to exploring alternative strategies. One such strategy is antibacterial photodynamic therapy (aPDT), a system that relies on light, oxygen, and a non-toxic dye (photosensitiser) to generate cytotoxic reactive oxygen species. This technique has already been shown capable of handling both drug-resistant and biofilm infections but has limited clinical approval to date, which is in part due to the low bioavailability and selectivity of hydrophobic photosensitisers. Nanotechnology-based techniques have the potential to address the limitations of current aPDT, as already well-documented in anti-cancer PDT. Here, we review recent advances in nanoparticle-based targeting tactics for aPDT.

**Graphical Abstract:**

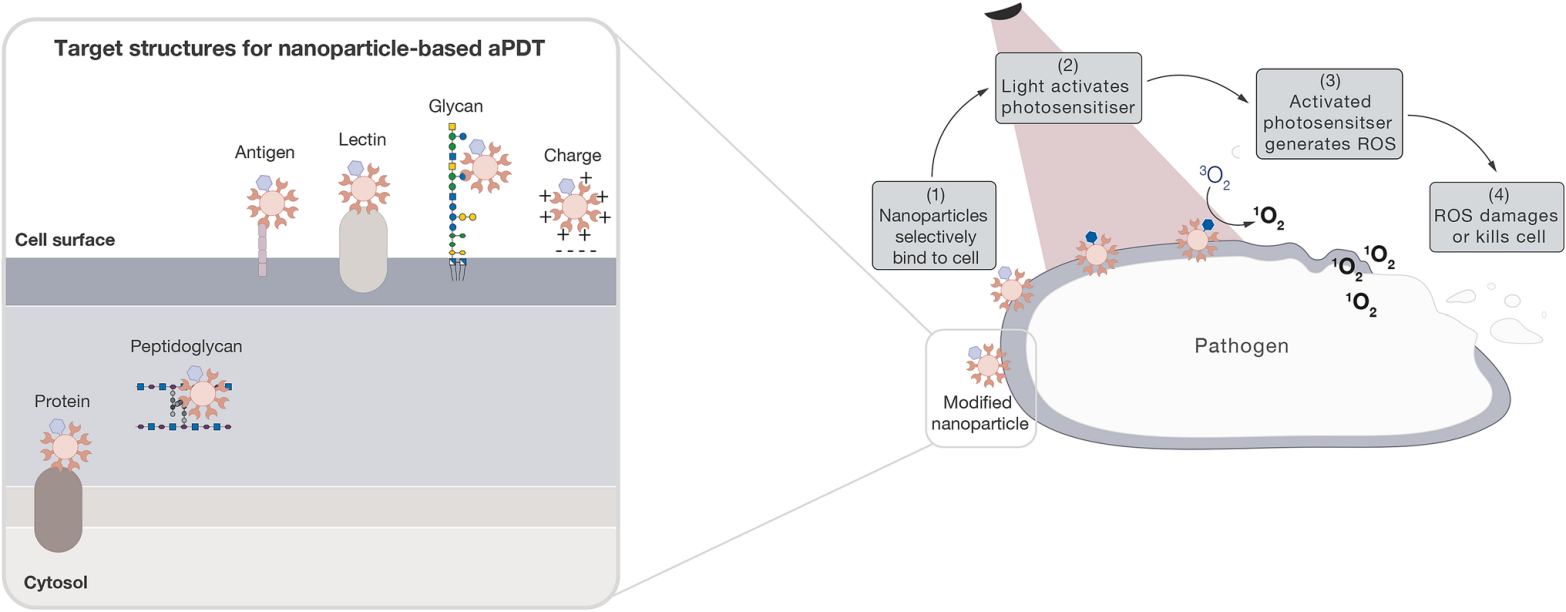

## Introduction

While antibiotics took a well-deserved centre-stage in research efforts during the twentieth century, the rise of drug resistance and lack of new antibiotics coming through to the clinics have left scientists urgently looking for alternatives to conventional treatments [1]. Using a variety of mechanisms, bacteria can be inherently resistant to antibiotics, developing drug resistance through mutations or acquiring drug resistance through horizontal gene transfer [2]. Common drug-resistance mechanisms used by bacteria include producing enzymes that degrade or modify the drug, upregulating drug efflux systems, and altering drug binding sites [3]. Many alternative antibacterial strategies are being explored to manage these drug-resistant infections, including antibacterial photodynamic therapy (aPDT)—a light-based therapy that could treat both drug-resistant and biofilm infections [4].

Although aPDT is often discussed as an alternative to antibiotics, the strategy actually predates antibiotic discovery, with Oscar Raab first demonstrating that PDT could kill microbial cells over 120 years ago [5]. The therapy relies on a non-toxic dye (photosensitiser), oxygen, and light to generate cytotoxic reactive oxygen species [6]. This light-dependency of PDT was first shown by Raab upon mixing acridine dye with protozoal cells (*Paramecium caudatum*), where cell death was witnessed in the presence of sunlight [5]. Subsequent work by Raab’s mentor, Hermann von Tappeiner, and others, showed that PDT is capable of tackling bacterial infections (e.g., lupus vulgaris caused by *Mycobacterium tuberculosis* and syphilis caused by *Treponema pallidum*) as well as skin cancer, and that PDT is an oxygen-dependent process, relying on reactive oxygen species for its destructive effects [7–11]. However, aPDT progress slowed following the discovery of penicillin in 1928, when antibiotics became the focus of antibacterial research efforts. aPDT began its revival in the 1970s, where its use for dental and skin infections were further probed, resulting in aPDT available in the clinic to treat acne vulgaris [12], typically targeting the opportunistic bacterium *Propionibacterium acnes* [13]. Many other infections are undergoing clinical trials with aPDT, such as dental, skin, and stomach infections, and many more potential applications are evident through in vivo and in vitro studies [14, 15]. Nonetheless, aPDT still faces challenges in selectivity and biocompatibility, but the field is actively developing methods to overcome these limitations. Here, we provide an update on ligand-selective targeting strategies (or ‘active targeting’) [16] currently being explored to treat bacteria with aPDT, with a focus on the use of nanoparticle technologies.

## Principles of aPDT

### Generating reactive oxygen species

PDT is a non-invasive therapy based on the combination of light, photosensitiser, and molecular oxygen [17]. In the presence of oxygen, the photosensitiser is activated with a particular wavelength of light, generating reactive oxygen species that cause cell damage and death [18]. Activated photosensitisers transfer either electrons (type I) or energy (type II) to surrounding oxygen-containing molecules to produce reactive oxygen species [19]—described in further detail below.

**Fig. 1.**
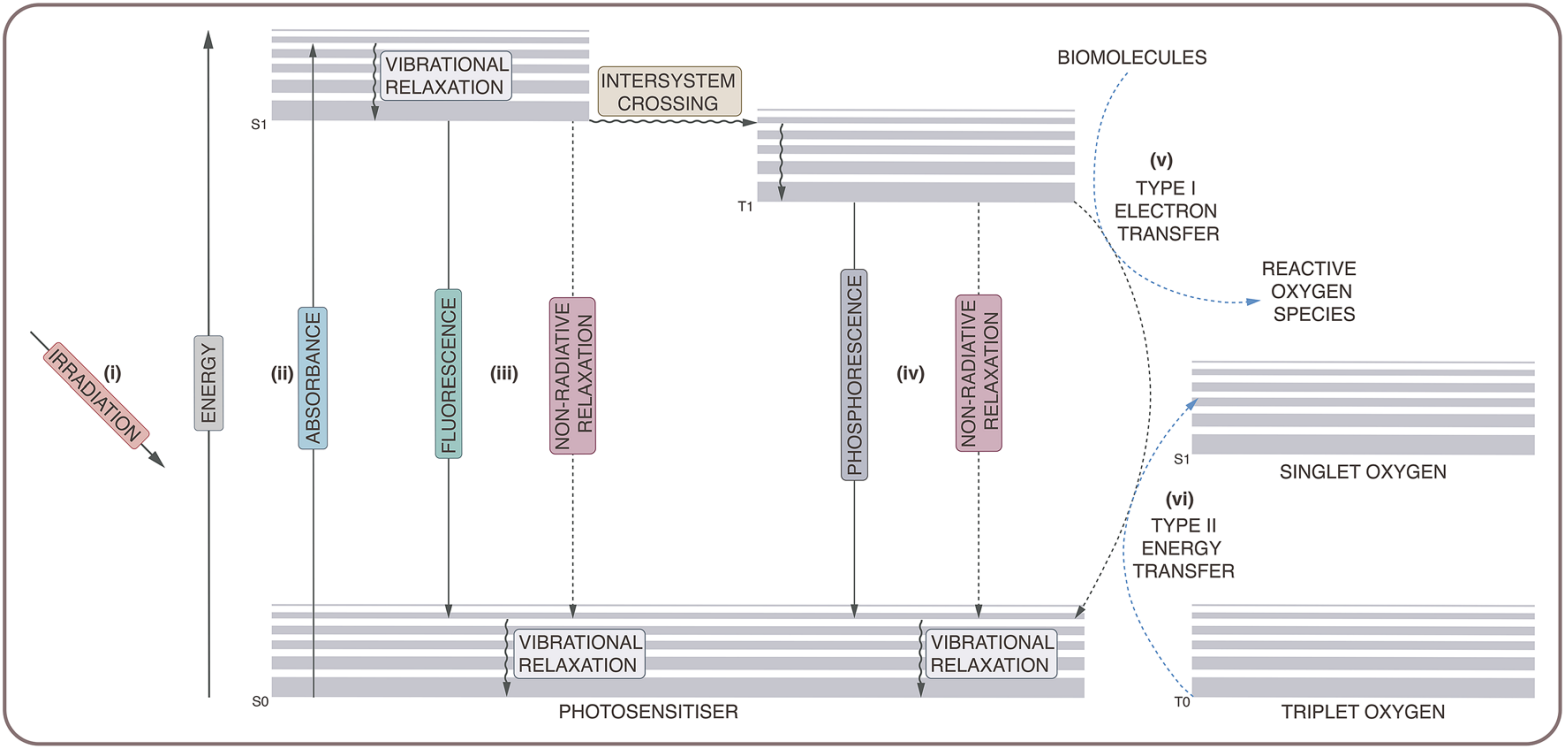
Jablonski diagram showing photosensitiser activation. Reactive oxygen species are generated through type I (v) and type II (vi) reaction mechanisms

Electrons may exist in the singlet state (paired electrons with antiparallel spin) or in the triplet state (unpaired electrons with parallel spin) [20]. Typically, molecules exist in the ground singlet state, where paired electrons occupy the lowest energy level (see Fig. 1). During activation, a photosensitiser absorbs energy from incident light (i), promoting the transition of ground state electrons to a higher energy level and placing the photosensitiser in an excited singlet state (ii) [21]. After a short time, the excited photosensitiser undergoes energy decay to return to the more energetically stable ground state. This energy dissipation occurs through a variety of processes (iii), including photon emission (fluorescence) or non-radiative means (vibrational relaxation, internal conversion). The excited photosensitiser may also undergo spin conversion into the more long-lived excited triplet state, through a process known as intersystem crossing [22]. Again, the excited molecule may return to the ground singlet state via photon emission (phosphorescence) or non-radiative processes (iv). However, while in the excited triplet state, the photosensitiser can also generate reactive oxygen species through interactions with oxygen-containing molecules via a type I or type II reaction [23].

As stated above, a type I reaction involves the electron transfer to oxygen or oxygen-containing biomolecules (v), producing free radical species, such as hydroxyl radicals, superoxide anions, and hydrogen peroxide [24]. A type II reaction involves the energy transfer from the excited triplet state photosensitiser to molecular oxygen (vi), generating singlet oxygen [21]. Although most molecules exist in the ground singlet state, molecular oxygen occurs in a ground triplet state. While in the excited triplet state, the photosensitiser can transfer energy to molecular oxygen, promoting electronic transition to an excited singlet state and producing highly reactive singlet oxygen [25].

### Photosensitiser selection for aPDT

**Fig. 2:**
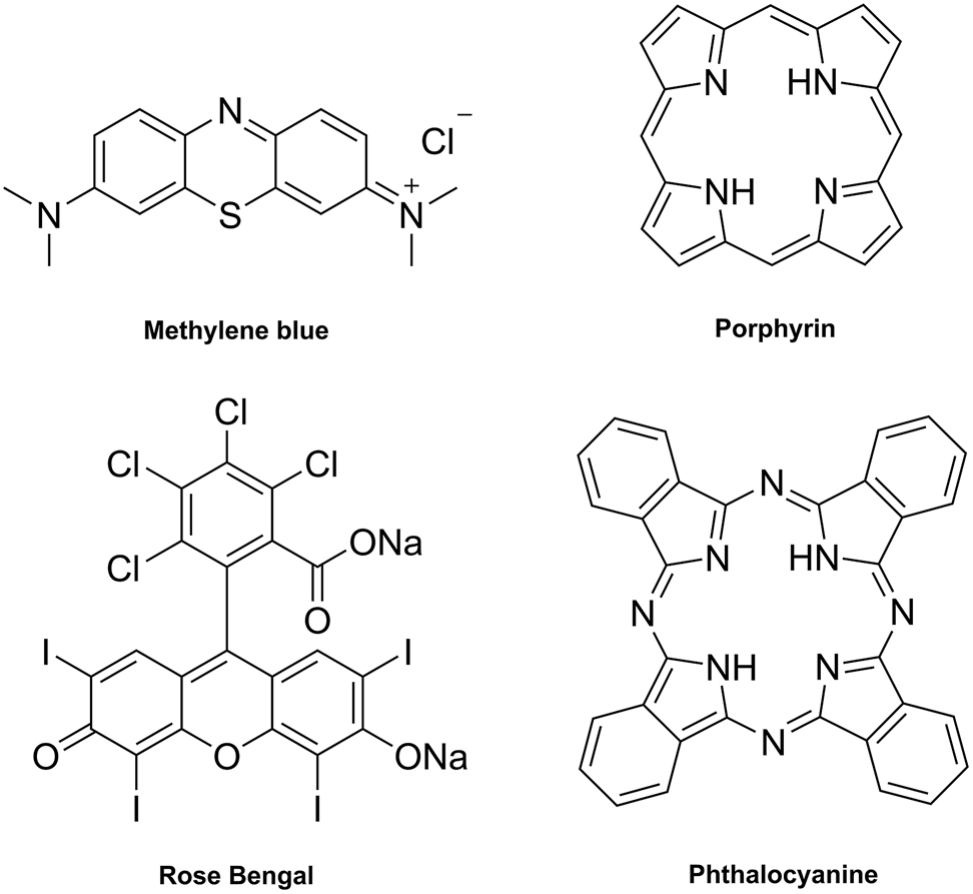
Chemical structures of the photosensitisers, methylene blue and Rose Bengal (left), used in aPDT, and chemical structures of tetrapyrrole photosensitiser core structures, porphyrin and phthalocyanine (right), used in aPDT

Photosensitiser physical, chemical, and biological properties make them optimal for medical purposes, including a large absorption extinction coefficient, long-lived excited triplet state, elevated triplet quantum yield, absence of dark toxicity, and rapid elimination from normal tissues to reduce side effects—although, photosensitisers do not necessarily fulfil these properties [26]. Most photosensitisers are highly conjugated structures, derivatised from either tricyclic aromatic dyes (such as methylene blue and fluorescein); tetrapyrroles (such as porphyrins and phthalocyanines). Examples of photosensitiser structures used in aPDT and of the tetrapyrrole core structures, porphyrin and phthalocyanine, are given in Fig. 2. Photosensitisers can be further classified as first, second, or third generation, depending on their properties and when they were discovered.

The first-generation photosensitisers are porphyrins that were derived from hematoporphyrin, including the first clinically approved photosensitiser: Photofrin® [27]. While still in use today, Photofrin® is a low-purity mixture of hematoporphyrin derivatives that are activated by short wavelengths of light (630 nm), leading to adverse clinical effects, such as prolonged photosensitivity, and limiting its clinical use, due to poor light penetration through tissue. Second-generation photosensitisers were designed to overcome these limitations by being activated at longer wavelengths of light, thus, allowing deeper tissue penetration, and having improved quantum yields and target-cell uptake [28]. Third-generation photosensitisers aim to improve delivery and uptake of photosensitisers to the target cells by introducing targeting structures (such as antibodies, glycans, and peptides), using nanoparticles as delivery systems, or—more recently—the combination of both (the focus of this review) [29]. Third-generation photosensitisers also include “theranostics”, which refer to systems that offer both diagnostic (e.g., by imaging) and therapeutic capabilities [30].

To date, only first- and second-generation photosensitisers have been clinically approved, and largely for cancer applications [31]. Plenty of studies have shown that these photosensitisers can eradicate both Gram-positive and Gram-negative bacteria, as well as biofilm infections—although Gram-negative bacteria are inherently less susceptible (see Sect. 2.3). For aPDT, photosensitisers typically bare cationic charges to improve uptake by bacteria due to anionic groups on the bacterial surface [32]. Among the most widely studied photosensitisers in aPDT are the tricyclic dyes: methylene blue and toluidine blue. These dyes are cationic, exhibit low toxicity, and successfully eradicate Gram-positive and Gram-negative bacteria. They have largely been used in accessible dental and skin infections, because they require short activation wavelengths [33, 34].

The majority of photosensitisers have poor water solubility due to their highly conjugated chemical structures, leading to aggregation after administration, which decreases their photodynamic activity [35]. Photosensitiser biocompatibility and targeted-cell uptake can be improved through direct molecular modifications, such as modifying photosensitisers with water-soluble glycans [36]. Alternatively, photosensitisers have been encapsulated or attached to the surface of nanoparticles, through either physical interaction or covalent coupling [37].

### Toxicity mechanism of aPDT

Photosensitisers have generally acted on the outer structures of bacterial cells, although intracellular uptake of activated photosensitisers can damage internal structures. To understand aPDT toxicity, we first need to consider the structural components of bacterial cells.

#### Structural sites of aPDT action

**Fig. 3:**
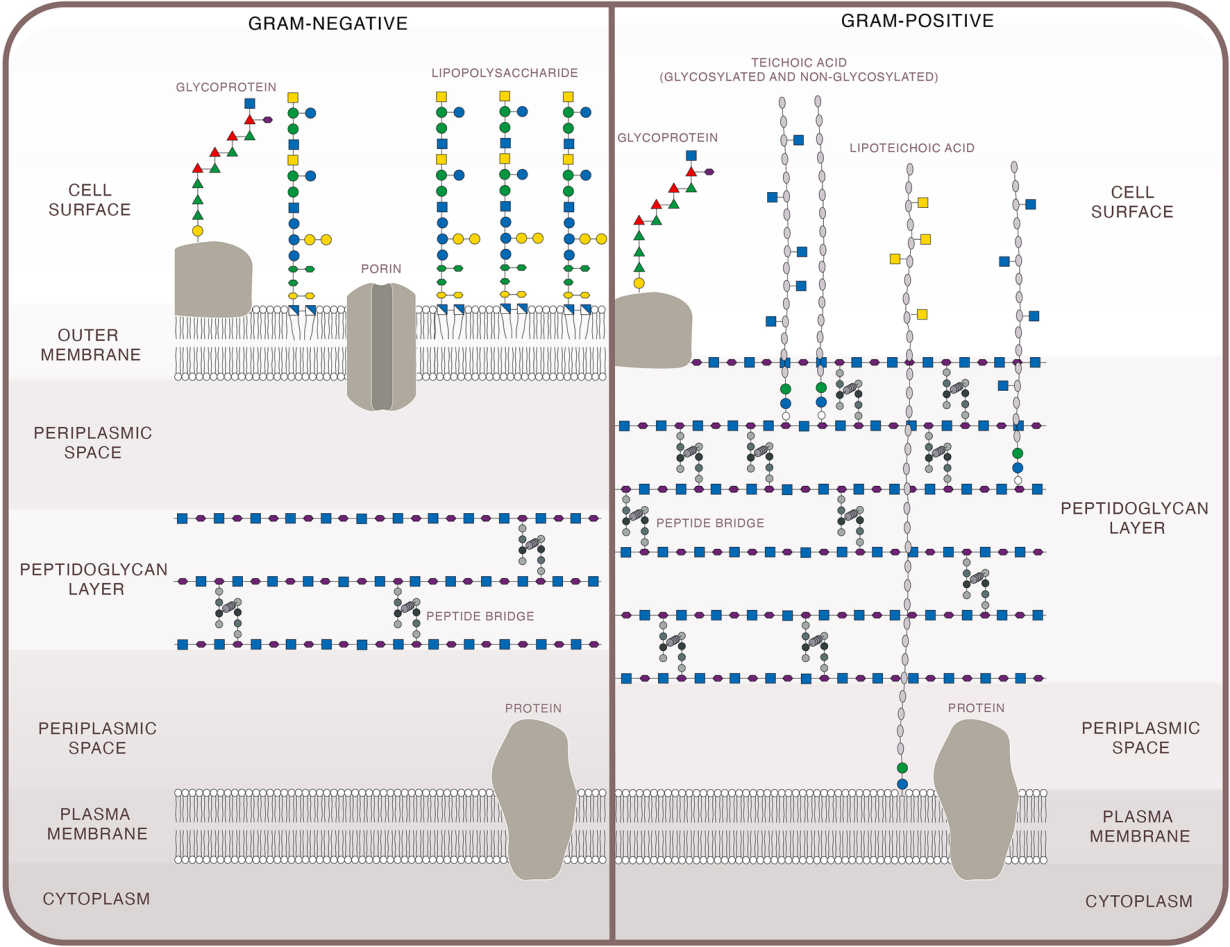
Illustration of key structural components of Gram-negative (left) and Gram-positive (right) bacterial cells

Differences in bacterial surface structures determine their drug susceptibility and drug targets. Although exceptions exist, bacteria can be loosely classified into Gram-positive and Gram-negative, based on structural differences in their cell walls (see Fig. 3) [38]. Both types share a peptidoglycan layer, composed of repeating units of *N*-acetylmuramic acid and *N*-acetylglucosamine that are cross-linked by peptide bridges [39]. Gram-positive species have a thicker peptidoglycan layer that is often populated with negatively charged polymers, such as teichoic acids [40]. Gram-negative peptidoglycan layers are thinner and have lower degrees of cross-linking, but are reinforced with an outer membrane containing lipopolysaccharide: a glycolipid that has non-exposed, conserved regions (lipid A) and exposed, variable regions (O antigen) [41]. This lipopolysaccharide layer surrounds the peptidoglycan and adds extra protection from external substances (such as drugs), making members of the Gram-negative group among the most extensively drug-resistant bacteria [42].

As stated above, exceptions to the structural classification do exist, for example, *Mycoplasma* do not produce peptidoglycan [43] and *Mycobacteria* share structural elements from both Gram-positive and Gram-negative bacteria [44]. The bacterial surface often has external protein appendages, such as fimbriae, pili, and flagella, which are needed for movement and adhesion [45]. The majority of bacterial species also have a glycocalyx, which is a carbohydrate coating used for attachment to and protection from the environment, such as attaching to host cells and avoiding phagocytic engulfment. A glycocalyx that is tightly associated with the bacterial surface is typically referred to as a ‘capsule’ and a more loosely associated glycocalyx is often referred to as a ‘slime layer’. All these external components have the potential to act as targets for aPDT systems [46].

In the body, bacteria may exist as a free-floating population of single cells (planktonic) or stationary, aggregated forms (biofilms). Biofilms are microbial communities (either single- or mixed-species) that are embedded in a secreted extracellular polymeric substance. The extracellular polymeric substance is made up of water, polysaccharides, proteins, enzymes, and DNA [47]. The composition of the extracellular polymeric substance varies between bacterial species and environment, for example, glucans and fructans have been identified in *Streptococcus mutans* (*S. mutans*) biofilms but Psl, Pel, and alginate have been found in *Pseudomonas aeruginosa* (*P. aeruginosa*) biofilms [48]. These extracellular polymeric substances link together to form a matrix that stabilises the biofilm, and can provide other benefits, such as trapping nutrients, restricting access from harmful substances, and permitting communication between cells [49, 50].

Biofilm cultures represent a huge clinical burden and are generally much harder to treat than their planktonic counterparts [51]. This is in part due to a biofilm’s extracellular polymeric substance, which offers a physical ‘barrier’ that restricts overall access to the bacteria, limiting drugs and immune system components from reaching their pathogen target [52, 53]. Many conventional antibacterial treatments attack bacterial membrane structures or need to be internalised to function [54] in metabolically active cells [55]. Not only is it difficult for drugs to physically reach the bacterial cell through the extracellular polymeric substance, but the extracellular polymeric substance also offers a medium for pathogens to share protective substances between each other, such as drug-resistance genes and drug-degrading enzymes or proteins [56]. Many bacteria also alter their gene expression under biofilm conditions that can slow their metabolic activity, reducing the effectiveness of conventional antibiotic toxicity mechanisms [57]. However, aPDT can overcome many of these limitations of conventional antibiotics when treating biofilm infections, which can be seen by looking at the aPDT mode of action.

#### aPDT mode of action

Regardless of the light activation mechanism (type I or type II), photosensitiser-generated reactive oxygen species exert cytotoxic effects on surrounding cells, where the damage is determined by the photosensitiser location, the molecular oxygen concentration, and the cell type. Depending on the medium, reactive oxygen species travel less than 150 nm, limiting their destructive properties to the immediate area [58]. Ideally, photosensitisers are activated by wavelengths of light between 600 and 800 nm, which can penetrate a maximum of 1 cm into tissue (depending on tissue type). This wavelength range is known in aPDT applications as the ‘therapeutic window’, since these wavelengths avoid strong absorption by biological molecules, such as deoxyhaemoglobin and water, and have sufficient energy to activate photosensitisers [59, 60].

Reactive oxygen species can destroy proteins, lipids, glycans, and genetic material (DNA/RNA), meaning that their cytotoxicity can impact all areas of cell biology, with damage largely dependent on the photosensitiser location [61]. Upon photosensitiser activation with incident light, reactive oxygen species oxidise membrane proteins and lipids, which creates cross-linking and pores to open up across the bacterial membrane, resulting in cell lysis. Alternatively, the photosensitiser can be taken up by the cell, where reactive oxygen species destroy intracellular components, such as DNA, proteins, and enzymes. As mentioned above, traditionally, aPDT has relied on photosensitisers reaching the outer or inner bacterial membranes due to a photosensitiser’s lipophilicity, leading to the assumption that the majority of cellular damage occurs on the outer parts of the cell.

With regard to biofilm clearance by aPDT, the current theory is that an ‘outside-in’ mode of action occurs. The photosensitiser enters the extracellular polymeric substance and, once activated with light, oxidises components of the extracellular polymeric substance, such as polysaccharides and proteins, breaking it down to expose the bacterial cells. The photosensitiser is then free to reach the bacterial cell surface, where it can induce cytotoxicity following the same mechanisms outlined above.

This widespread reactive oxygen species attack on bacteria reduces bacterial ability to develop resistance against the aPDT toxicity mechanism, as it is unclear, where the attack is originating. Although antioxidant enzymes exist, no enzyme has yet been documented to target singlet oxygen (the reactive oxygen species responsible for the most effective bacterial damage) [21, 62]. However, bacteria may develop resistance by increasing their efflux pump expression, expelling the photosensitiser from the cell and reducing damage. Although many classes of photosensitisers have been assessed, the only class that has been shown to be removed by efflux pumps are the phenothiazinium dyes [6, 63]. Furthermore, the photosensitiser remains intact after activation, allowing the photosensitiser to be recycled for further excitation and generation of reactive oxygen species [64]. In addition, reactive oxygen species damage is catalytic, where the initial generated reactive oxygen species yield further radical products, causing additional cellular damage [27]. Consequently, in contrast to antibiotics, aPDT damage is amplified and pervasive.

While research is in its infancy, there is evidence that aPDT can stimulate the immune system to improve bacterial clearance. For example, Tanaka et al*.* reported that the innate immune system was stimulated after aPDT of an in vivo bacterial (methicillin-resistant *Staphylococcus aureus* (MRSA)) arthritis mouse model, where neutrophil stimulation and recruitment to the infection site were critical for bacterial clearance [65]. Immune stimulation is an already well-established mechanism in anti-cancer PDT. Here, PDT-killed cancer cells release innate immune system-stimulating factors (cytokines, chemokines, damage-associated molecular patterns), which direct clearance of the cancer cells and can even generate an adaptive immune response [65, 66]. By stimulating an immune response towards the infection site, it is hoped that the immune system can tackle infections elsewhere in the body and that lower doses of PDT treatment are required. During aPDT inactivation, pathogens can release damaging substances on their break down, such as toxins and lipopolysaccharides. However, aPDT has been shown to neutralise and destroy these toxic substances too, reducing toxicity to the host [67].

## Overcoming aPDT limitations with nanoparticles

Currently, aPDT is restricted to areas where current light systems can access and emit sufficient light intensities to activate the photosensitiser, meaning aPDT is limited to localised and superficial infections, such as skin and dental infections. In addition, aPDT can exhibit low selectivity for target cells and the necessary reagents can also have low bioavailability [68]. Many known photosensitisers rely on structure and charge to achieve specificity for the target pathogen. The low selectivity of aPDT can also cause damage to the microbiota, such as the gut, mouth, and skin, yielding secondary opportunistic infections [69, 70]. As mentioned above, photosensitiser bioavailability is further restricted by its hydrophobicity; hydrophobic drugs can aggregate in the body and are rapidly cleared by the immune system through complexation with plasma proteins and opsonisation [71, 72], reducing their circulation time and biocompatibility.

With the advancement of light delivery technologies, such as optical fibre systems, [73] aPDT may be expanded for use in deeper-seated infections. For example, photosensitiser could be administered into the body, accumulating at the site of infection after an optimal period, and irradiated with light that is delivered to the site through fibre optic systems—a strategy that has already been demonstrated in anti-cancer PDT (such as pancreatic cancer) [74]. However, without developments in strategies for effective delivery of light across the body, aPDT is unable to be applied to systemic infections. With this limitation in mind, this review focuses on the targeting strategy rather than treatment application.

Overcoming the biocompatibility and selectivity limitations of aPDT is needed for the photosensitiser to selectively bind to pathogens over host cells and the microbiota, particularly in applications where the photosensitiser needs to accumulate at the infection site, such as after systemic administration in clinical aPDT [75]. To address these limitations, scientists have altered photosensitiser properties through direct chemical modifications (such as introducing cationic charge) and/or have used nanoparticles as photosensitiser delivery systems. Nanoparticles are between 1 and 100 nm in size and available in a range of shapes, including spherical, rod, square, triangle and star [76]; although, in this review, particles with a size < 1000 nm have been included as nanoparticles. There are many options available for the nanoparticle material in aPDT (see Yin et al*.* [68]), loosely classified into organic and inorganic, each offering their own advantages and limitations. An advantage shared by all nanoparticles is their high surface area to volume ratio, which allows for high drug (photosensitiser) loading. Their size can create distinctive electronic and optical properties and can be fine-tuned to improve uptake across the cell membrane and the blood–brain barrier. By modifying the nanoparticle material and/or surface, improvements can be made to the bioavailability and biocompatibility of a drug, as well as its selectivity. These surface modifications can involve multiple types of ligands, incorporating different properties, such as targeted photosensitiser delivery.

**Fig. 4:**
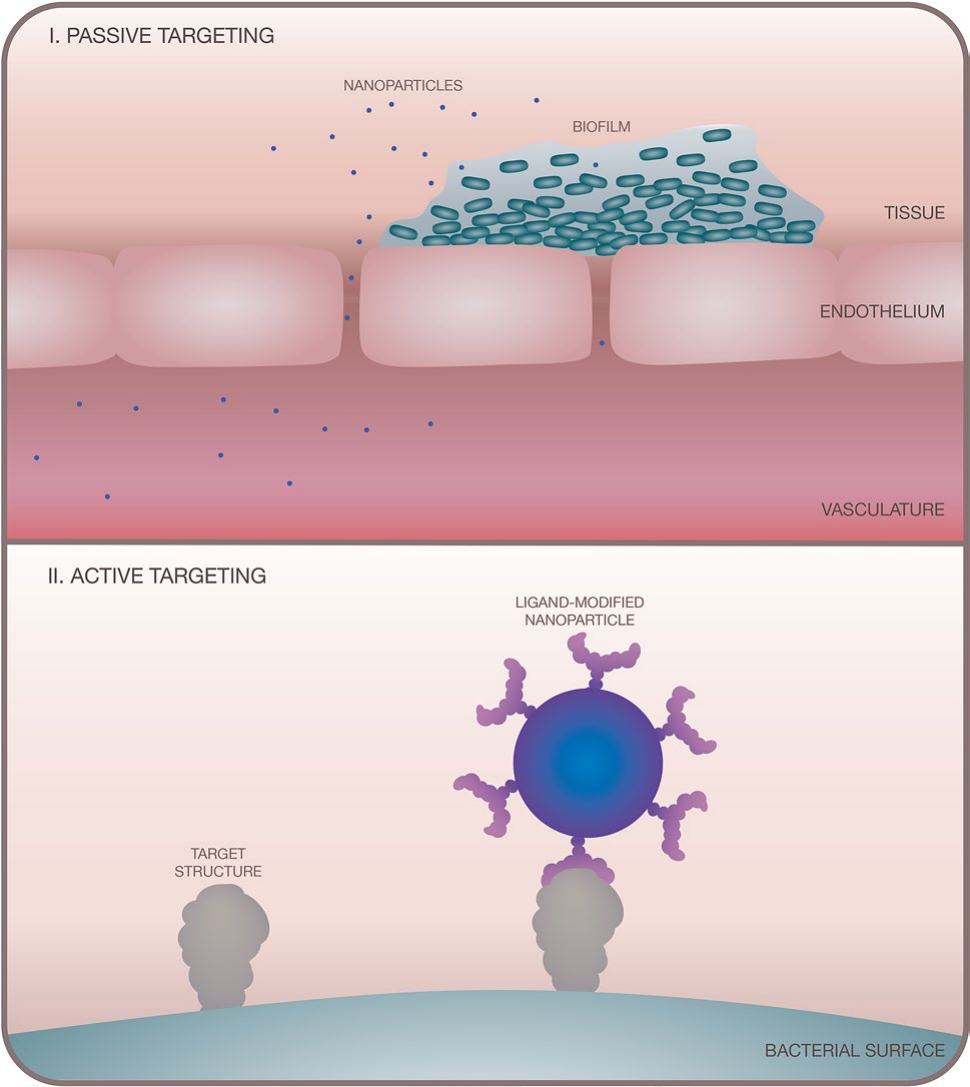
Illustration showing (I) passive targeting and (II) active targeting approaches using nanoparticles in aPDT

The approaches used for nanoparticle-based targeting in aPDT can be split into ‘passive’ or ‘active’. Passive targeting relies on prolonged drug circulation in the body and preferential accumulation of the drug to the infection site over other areas of the body (see Fig. 4-I). Active targeting relies on ligand–receptor selective binding to the target bacterial cell (see Fig. 4-II) [77].

With regards to passive targeting, nanoparticles with appropriate size, shape, material, and surface modification are well-documented for their ability to breach leaky vasculature associated with disease and inflammation (known as the ‘enhanced permeation and retention (EPR) effect’) and for their potential to prolong drug circulation time in the bloodstream [78]. While healthy vasculature forms an effective barrier, inflammation causes the vasculature surrounding the infection site to become more permeable. This increased permeability allows immune cells to infiltrate the area and permits nanoparticles to access and accumulate at the infection site, due to nanoparticle size and poor clearance [79]. The EPR effect has been well exploited in nanoparticle-based systems for anti-cancer PDT, but less so in aPDT. Consideration should be given to the timing of treatment application in passive targeting approaches for aPDT as vasculature loses permeability at later stages of infection, so the EPR effect is limited to earlier stages of acute infection [80]. As well as restricting the timing of treatment application, passive targeting lacks selectivity for the bacterial cell and can result in host toxicity and damage to the microbiota.

## Recent advances in nanoparticle-based active targeting approaches in aPDT

Active targeting requires ‘targeting ligands’ that selectively recognise structures on pathogens. As nanoparticle surfaces can be modified with multiple types of ligands, they offer an easy method of combining targeting ligands and photosensitisers for an active targeting aPDT approach [81]. These targeting ligands can come in different forms, offering low selectivity (such as charge) to high selectivity (such as antibodies, peptides and glycans).

### Low-selectivity nanoparticle-based targeting in aPDT

We define the following low-selective targeting systems as strategies that provide selective binding to bacterial cells over human cells or bind under infectious environmental conditions (such as pH change). Different strategies have been employed for this task, but this review will focus on charge and antibiotics for low-selective approaches. For a summary of experimental conditions and the following studies, please see Table 1.

**Table 1:** Summary of the nanoparticle-based targeting approaches in aPDT discussed in this review

Targeting agent	Ref	Target species	In vitro or in vivo PDT treatment^a^	Nanoparticle		Photosensitiser	Targeting structure	Irradiation conditions			
				Material	Size/nm			Light source	Time/min		Irradiance/mW/cm^2^
Charge	[85]	*E. coli, S. aureus*	In vitro	Polymer	78–90	Chlorin e6	Charge (pH responsive)	660 nm laser		10	100
	[86]	*E. coli, N. gonorrhoeae*	In vitro	Zeolite L-based	50	SiIV-phthalocyanine dihydroxide and N,N’-bis(2,6-dimethylphenyl) perylene-3,4,9,10-tetracarbodiimide	Amino ligand	570–900 nm tungsten lamp		150	3
	[88]	MRSA*, P. aeruginosa*	In vitro*,* planktonic	Liposomes	136.6 ± 1.6	Temoporfin	Antimicrobial peptide (WLBU2)	652 nm laser		1.7 (100 s)	1000
	[89]	*S. aureus*	In vivo*,* mouse thighs	Manganese oxide (coated with BSA)	50 ± 10	Indocyanine green	Antimicrobial peptide ubiquicidin29-41 and antibiotic gentamicin	808 nm laser		15	100
Antibiotic	[90]	MRSA*,* methicillin-sensitive *S. aureus*	In vitro, planktonic; in vivo*,* rats	Gold	79 ± 43	Plasmon gold nanoparticle	Amoxicillin	White light LED		30	0.6^b^
	[92]	*P. aeruginosa*, *S. aureus*	In vitro, biofilm	Gold	79 ± 43	Plasmon gold nanoparticle	Amoxicillin	White light LED		60–180	Red = 5.5, Red = 9.9, Blue = 2.2, White = 0.3
	[94]	Vancomycin-resistant *E. faecium* and *E. faecalis*	In vitro*,* planktonic; in vivo*,* mouse wounds	Gold, silver, silica	110	2,3-naphthalocyanine	Vancomycin	780 nm laser		30	30
	[95]	Vancomycin-resistant *E. faecium* and *E. faecalis*	In vitro*,* planktonic; in vivo*,* mouse wounds	Copper sulfide	15 ± 5	Copper sulfide nanoparticle	Vancomycin	980 nm laser		10	0.8
	[96]	Clinical isolates of MRSA, vancomycin-resistant *E. faecalis*, *E. faecalis, S. aureus*, *B. cereus*, methicillin-sensitive *S. aureus*, *E. coli* O157:H7:K, *S. typhimurium*	In vitro*,* planktonic	Iron oxide	400 ± 19	t-PtCP ([5,15-bisphenyl-10,20-bis(4-methoxycarbonylphenyl)-porphyrin] platinum)	Vancomycin	510 nm laser		0–60	6.1
	[97]	*E. coli*, methicillin-sensitive *S. aureus*, MRSA	In vitro, planktonic and biofilm; in vivo*,* rabbit endophthalmitis	Zeolitic imidazolate framework-8-polyacrylic acid	150	Methylene blue	Vancomycin	630 nm laser		5	202
Glycans	[102]	*P. aeruginosa* and *S. aureus*	In vitro, planktonic	Silver nanoparticles	162.6 ± 3.6	BODIPY	Galactose	400–800 nm laser		15	1.5
	[103]	Drug-resistant *P. aeruginosa*	In vitro, planktonic and biofilm; in vivo*,* mouse lung model for pneumonia	Gadofullerene-based	171—187	Indocyanine green	Galactose and/or fucose	808 nm laser		5	2000
	[105]	*P. aeruginosa* and *S. aureus*	In vitro*,* planktonic	BODIPY core and 2-(dimethylamino)ethyl	16–18^c^	BODIPY	Galactose	400–800 nm LED		5	25
	[106]	*B. subtilis* and *E. coli*	In vitro*,* planktonic	Poly-5,15-diphenyl(2,5’-dithienylen)-10,20-di(3,5-di-O-TEG-phenyl)	50–80	Porphyrin	Acetylated glucose	White light LED		10	22
	[107]	*S. aureus*	In vitro, planktonic	Hyperbranched polyglycerol	19.5^c^	5,10,15-tris(3-hydroxyphen- yl)-20-[4-(prop-2-yn-1-ylamino)tetrafluorophenyl]porphyrinato}-zinc(II)	Mannose	652 nm laser		85^d^	100^e^
	[108]	*E. coli*, *P. aeruginosa*	In vitro, planktonic	Mesoporous silica	180–200	Methylene blue	Mannose	652 nm LED		30	16, 32^e^
	[111]	*P. aeruginosa*	In vivo*,* rabbit keratitis and mouse meningitis models	Acrylamide-based polymeric	38	Methylene blue	Imprinted structure of * P. aeruginosa* lipopolysaccharide	650 nm laser		20	125
Antibody	[113]	MRSA	In vitro*,* planktonic; in vivo*,* mouse wounds	Iron oxide	103 ± 7	Hematoporphyrin	Anti-MRSA antibody	517 nm LED		60	3.9 (in vitro), 11.8 (in vivo)
	[114]	MRSA and methicillin-sensitive *S. aureus*	In vitro, planktonic	Gold (coated with BSA)	1.8 ± 0.4^f^	Photosens™	Anti-*Staphylococcus aureus* antibody	660 nm LED		0–60	25
	[115]	Multi-drug resistant *Salmonella* (DT104)	In vitro*,* planktonic in rabbit blood	Iron core, gold shell	70	Methylene blue	Anti-*Salmonella* DT104 antibody	670 nm laser		12	1000–2000

^a^Other studies may have been reported in the study but only the culture form for aPDT application is detailed in the table

^b^Energy reported in power (W)

^c^Nanoparticle size reported in molecular weight (kDa)

^d^Time reported in seconds (s)

^e^Energy reported in fluence (J/cm^2^)

^f^Measurement of gold core with TEM

#### Charge

Charge has been extensively explored as a targeting strategy in aPDT. As stated above, many of the photosensitisers used in aPDT bare cationic charges to provide preferential binding to bacterial cells over human cells [82]. This preferential binding is due to the cationic photosensitiser localising to anionic groups on the bacterial surface (such as teichoic acids and lipopolysaccharides) [83]. While modifying free photosensitisers with a cationic charge can improve localisation to a bacterial cell, using nanoparticles can increase the uptake of photosensitisers and reduce clearance from bacterial cells and biofilms (through pumps) compared to free photosensitisers [84]. Recent reports have developed cationic nanoparticles, either through the nanoparticle material itself being cationic or through the nanoparticle surface being modified with cationic ligands. For example, Liu et al*.* [85] targeted *Escherichia coli *(*E. coli*) and *S. aureus* using pH-responsive nanoparticles, where the nanoparticle material became cationic at around pH 6.0. These nanoparticles were polymeric, containing chlorin e6 as the photosensitiser. The zeta potential changed from − 1.45 mV at pH 7.4 to + 11.6 at pH 6.0, as the polymer became protonated. The authors reported a size change from 78 nm (at pH 7.4) to 90 nm when the pH was decreased to 6.0. The aPDT effect of these nanoparticles was investigated with irradiated in vitro bacterial cultures at two different pH values (6.0 vs. 7.4). The aPDT treatment was found to be pH-dependent, as complete eradication of both bacterial species was only observed at pH 6.0.

Instead of relying on the nanoparticle material being charged, Strassert et al*.* modified the nanoparticle surface with cationic ligands [86]. Inorganic nanoparticles (zeolite L-based, 50 nm) were coated with two photosensitisers (SiIV-phthalocyanine dihydroxide and *N*,*N*′-bis(2,6-dimethylphenyl) perylene-3,4,9,10-tetracarbodiimide) and amino ligands to target *E. coli* and *Neisseria gonorrhoeae* (*N. gonorrhoeae*) cultures. These cationic nanoparticles (zeta potential + 9.8 ± 1.5 mV) eradicated both in vitro bacterial cultures after irradiation. Scanning electron microscopy (SEM) images of *E. coli* cultures mixed with the particles confirmed the localisation of the nanoparticles on the bacterial membrane.

Another charged-based ligand that has been explored in targeted aPDT are cationic antimicrobial peptides, which are small molecules (10–50 amino acids) [87]. Cationic antimicrobial peptides offer straightforward synthesis and ease of nanoparticle functionalisation, with a common nanoparticle functionalisation strategy in aPDT being to covalently couple peptides onto the surface of nanoparticles through EDC-mediated cross-linking chemistry. Yang et al*.* used liposomes (*ca.* 140 nm) modified with photosensitiser (Temoporfin, m-THPC) and antimicrobial peptide (WLBU2) to target *P. aeruginosa* and *S. aureus* [88]. The nanoparticles functionalised with the cationic antimicrobial peptide were incubated with in vitro bacterial cultures and irradiated: *S. aureus* cultures were completely eradicated (90 min) and *P. aeruginosa* culture viability was reduced by *ca.* 2000-fold (180 min), with no dark toxicity (i.e., no toxicity was observed in the absence of light).

**Fig. 5:**
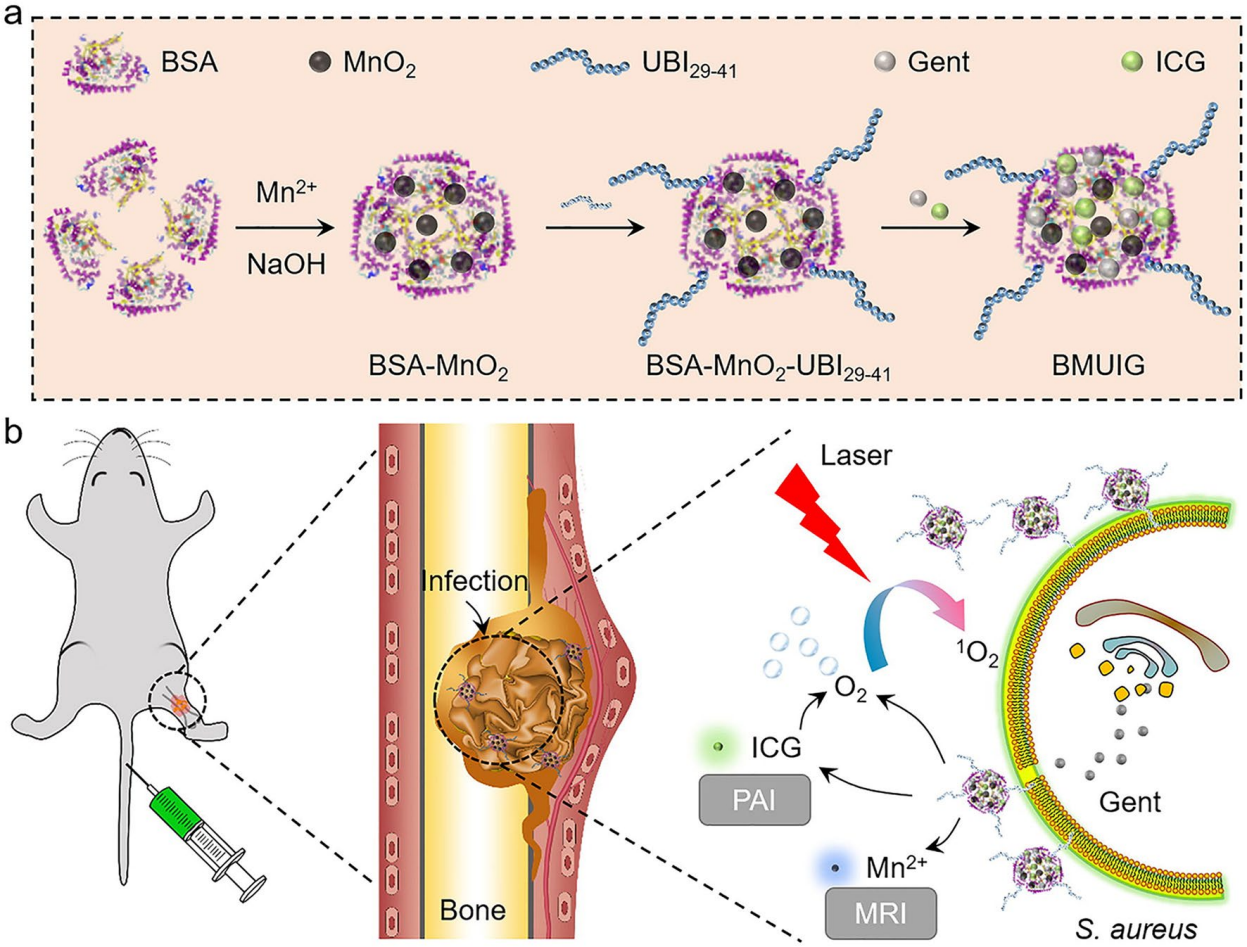
Schematic representation of **a** the synthesis of BSA-coated manganese oxide nanoparticles modified with the antimicrobial peptide ubiquicidin 29-41 (UBI_29-41_), gentamicin (Gent) and indocyanine green (ICG); and **b** nanoparticle application in mice for imaging and photodynamic therapy. Reprinted from Ref. [89] with permission from Elsevier^§^

Following a similar approach, Lu et al*.* modified bovine serum albumin (BSA)-coated manganese oxide nanoparticles with the antimicrobial peptide ubiquicidin 29-41 (UBI_29-41_) and the antibiotic gentamicin (Gent) to target *S. aureus* (see Fig. 5) [89]. The particles also contained indocyanine green (ICG) as the photosensitiser. Using photoacoustic imaging (PAI), the peptide-modified nanoparticles were shown to localise to in vivo* S. aureus*-infected mouse thighs (osteomyelitis model) with a higher signal than that of mice treated with the free photosensitiser. Following irradiation, micro-computed tomography imaging showed a reduction in inflammation at the infection sites of mice treated with the nanoparticles.

While cationic nanoparticles have shown much potential in improving aPDT effectiveness, charge generally relies on widespread and non-specific binding interactions with the bacterial surface, with potential to bind many different types of microbial cells. Therefore, as mentioned above, cationic nanoparticles may also bind and damage non-target microorganisms, such as members of the microbiota.

#### Antibiotics

Although their effectivity is becoming more limited, attaching broad- or narrow-spectrum antibiotics (classified by the range of bacterial species they target) to nanoparticles enhances drug efficacy by improving delivery to the target bacteria [90–92]. Consequently, antibiotic–nanoparticle combinations offer a viable technique for selective aPDT towards bacteria.

Silvero et al*.* used gold nanoparticles modified with amoxicillin to target methicillin-sensitive and MRSA strains, where the gold nanoparticles functioned as both the scaffold and the photosensitiser [90]. Amoxicillin is a β-lactam bactericidal antibiotic that targets penicillin-binding proteins. These proteins are responsible for catalysing transglycosylation (polymerisation) and transpeptidation (cross-linking) of the peptidoglycan layer. Amoxicillin inhibits the transpeptidase activity, leading to cell lysis by activating autolytic enzymes and osmotic swelling [91]. Silvero et al*.* selected gold nanoparticles as these particles improve delivery and circulation time, are easy to functionalise, and can function as a photosensitiser [90]. The amoxicillin-modified gold nanoparticles eradicated both in vitro* S. aureus* cultures after irradiation, with minimal dark toxicity. The particles had excellent biocompatibility with human cells upon PDT treatment and good distribution and clearance in animal studies (rats), where transmission electron microscopy (TEM) images demonstrated that the particles were detected in organs after 5 h and completely cleared from the body after 24 h, with normal tissue histopathology. In contrast, uncoated gold nanoparticles were not found in urine samples and were only detected at low levels in the body after 2 h which could indicate that uncoated AuNPs are neither well distributed in the body nor eliminated by the renal excretion. The results reported in this paper suggest that amoxicillin coating stabilises the resulting nanoparticles and provides the surface charge and surface chemistry required for renal clearance.

Following on from these findings, Rocca et al*.* used the same amoxicillin-modified gold nanoparticles to target biofilm cultures of two strains of *P. aeruginosa* and *S. aureus* [92]. As shown in Silvero et al*.* [90], particles in a sample were polydisperse, varying in size (79 ± 43 nm) and shape (spherical, triangular, hexagonal, and nanorods), and an LED array was used to irradiate the samples to accommodate the wide absorption wavelengths of these particles. Compared to untreated samples, irradiated particles caused a significant reduction in *P. aeruginosa* (*ca.* 70%) and *S. aureus* (50–70%) biofilm viability, with no dark toxicity observed. Imaging studies showed that these particles destroyed the biofilm matrix and reduced cell surface adhesion. It is important to mention that size polydispersity has an influence on the antimicrobial PDT effect with different shapes causing different degrees of bacterial and biofilm damage and different sizes behaving differently when diffusing into the extracellular polymeric substance of the biofilm matrix [92].

**Fig. 6:**
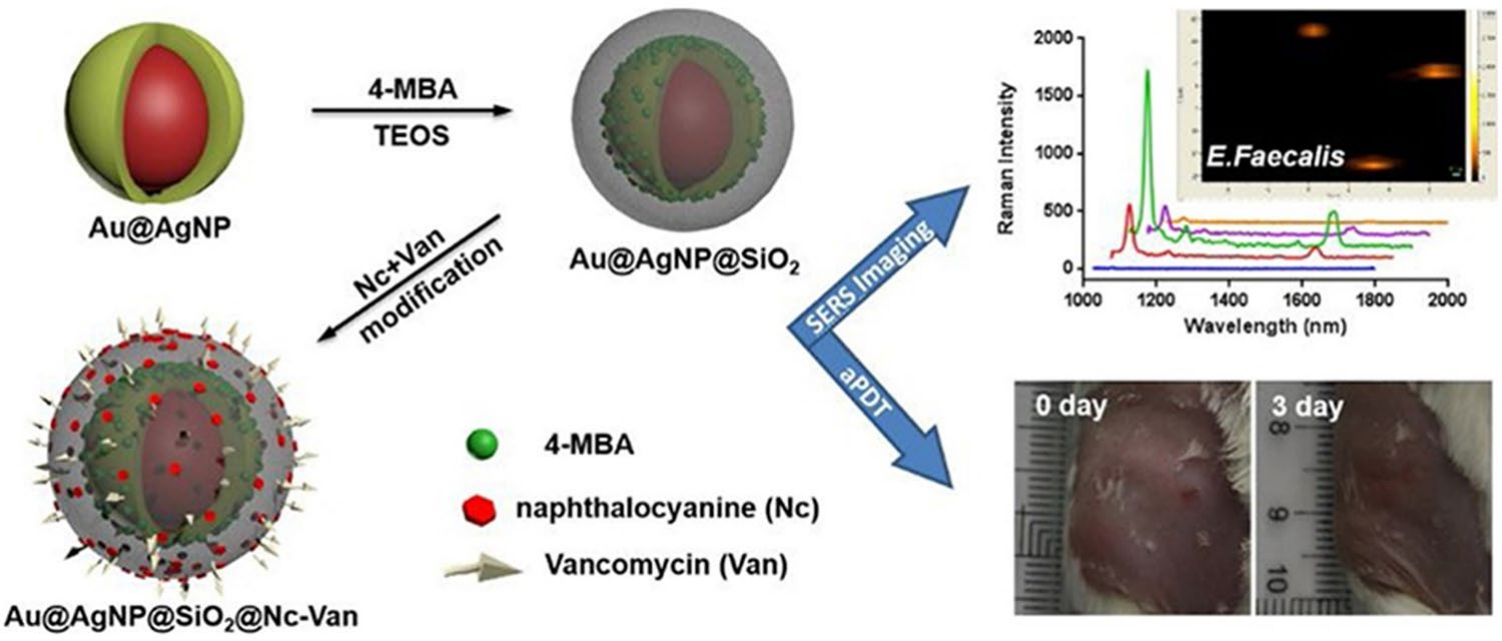
Schematic representation of the modification route of silver-coated gold nanoparticles (Au@AgNP) that were further modified with silica, naphthalocyanine, and vancomycin, which were used for SERS imaging and aPDT. Reprinted from Ref. [94] with permission from Elsevier^§§^

Another antibiotic that has been used in aPDT is vancomycin, which targets Gram-positive bacteria by inhibiting peptidoglycan synthesis. As with all antibiotics, vancomycin is becoming increasingly ineffective against bacteria due to emerging drug resistance [93]. However, Zhou et al*.* looked to vancomycin to develop targeted nanoparticles (110 nm) against vancomycin-resistant strains: *Enterococcus faecium* (*E. faecium*) and *Enterococcus faecalis* (*E. faecalis*) [94]. While these strains may not be sensitive to vancomycin as mutations in their peptidoglycan structure prevent vancomycin from inhibiting peptidoglycan synthesis, vancomycin can still access and attach to the peptidoglycan layer of these drug-resistant strains. Consequently, providing that vancomycin can still bind to peptidoglycan, it can still be used for targeting, but not eradicating, the bacteria. Nanoparticles consisted of a gold core coated in silver and silica, which were then modified with photosensitiser (2,3-naphthalocyanine dihydroxide) and vancomycin (see Fig. 6). The nanoparticle core generated surface-enhanced Raman scattering (SERS) signals, which were used to identify bacterial infections. As the photosensitiser is activated with near-infrared wavelengths, infections can be targeted that are deep-rooted or thicker in size. The nanoparticles were tested against Gram-positive (vancomycin-resistant enterococci and vancomycin-sensitive *Bacillus subtilis* (*B. subtilis*)) and Gram-negative (*E. coli*) bacteria. Using the core-generated SERS signal, the nanoparticles were shown to selectively bind to the cell walls of the Gram-positive strains tested. Vancomycin-modified nanoparticles showed higher antibacterial activity against the drug-resistant enterococci strains (*E. faecium* and *E. faecalis*) compared to nanoparticles without vancomycin, in vitro. The irradiated vancomycin-modified nanoparticles significantly reduced *E. faecalis* cell count in animal studies (mouse wounds), providing a 1,000,000-fold reduction in bacterial cell count compared to particles that were not irradiated and exhibited higher in vivo antimicrobial activity towards *E. faecalis* than nanoparticles without vancomycin. The particles also demonstrated good biocompatibility with limited damage to human skin cells (HaCaT cell line) [94].

Zou et al*.* also used vancomycin to target vancomycin-resistant *E. faecium* and *E. faecalis* strains using vancomycin-modified copper sulphide nanoparticles (*ca.* 15 nm), which act as both the photosensitiser and the scaffold [95]. The particles are activated at long wavelengths (> 900 nm), targeting deep-seated infections and also exert photothermal therapy (PTT) effects through localised heating. Both the PDT and PTT capabilities of the copper sulphide were demonstrated, leading to an overall ‘photokilling’ effect on the bacteria. Irradiated particles eradicated (> 99%) enterococci strains in in vitro studies, with no significant dark toxicity. The particles were assessed for in vivo aPDT effectivity against vancomycin-resistant enterococci-infected mouse wounds. Through visual evaluation of the wound and histological examination (hematoxylin and eosin stained), irradiated, nanoparticle-treated mouse wounds had reduced signs of infection (such as abscess regression and reduced inflammation) compared to non-irradiated mice wounds.

Choi et al*.* developed multifunctional iron oxide magnetic particles (*ca.* 400 nm) for targeted eradication of bacteria, including Gram-positive (*E. faecalis*, *S. aureus*, and *Bacillus cereus* (*B. cereus*), methicillin-sensitive *S. aureus* and vancomycin-resistant enterococci) and Gram-negative (*E. coli* and *Salmonella typhimurium* (*S. typhimurium*)) strains [96]. Vancomycin effectiveness is usually restricted to Gram-positive strains, as vancomycin cannot reach the peptidoglycan layer due to the low permeability of the outer Gram-negative bacterial membrane. However, Choi et al*.* [96] developed iron oxide particles that can target Gram-negative strains by improving the delivery of vancomycin through the outer membrane. Iron oxide particles were modified with the photosensitiser [5,15-bisphenyl-10,20-bis(4-methoxycarbonylphenyl)-porphyrin] platinum (t-PtCP) and vancomycin. The magnetic particles were able to capture the bacteria and, due to their high level of saturation magnetisation and superparamagnetism, the bacteria–particle conjugates could be removed from the bacterial solution by a magnet. The capturing capacity of the functionalised magnetic particles was 10^6^ CFU/mL for Gram-positive bacteria and 10^5^ CFU/mL for Gram-negative bacteria. Through TEM analysis, the vancomycin-modified nanoparticles were found to bind to the cell membranes of *S. typhimurium*, *E. faecalis*, *B. cereus,* methicillin-sensitive *S. aureus,* MRSA, and vancomycin-resistant enterococci. After irradiation, vancomycin-modified nanoparticles completely eradicated in vitro Gram-positive (6 min) and Gram-negative (1 h) bacterial cultures and had good biocompatibility with the L-929 murine fibroblast cell line.

Chen et al*.* designed a pH-responsive metal–organic framework (zeolitic imidazolate framework-8-polyacrylic acid) that was loaded with methylene blue, coated with silver nanoparticles and then modified with vancomycin to eradicate *E. coli*, *S. aureus* and MRSA [97]. The synthesis yielded spherical nanoparticles (*ca.* 150 nm), which released methylene blue in low pH (5.5) environments due to structural changes (protonation leading to swelling and collapse) in the metal–organic framework. The vancomycin-modified particles were effective at treating both planktonic and biofilm bacterial cultures, which were completely eradicated after irradiation, and show superiority over particles without vancomycin. Control particles that lack the silver component but contained the photosensitiser drug only showed toxicity against the bacterial cultures upon irradiation. This antimicrobial efficiency was reduced compared to metal–organic frameworks loaded with methylene blue and coated with silver nanoparticles, which the authors suggest is likely due to the presence of the antibacterial silver nanoparticles. However, the nanoparticles were compatible with mammalian cell lines (retinal pigment epithelial cell line and human corneal epithelial cell line) when used at concentrations less than 10 μg/mL and were subsequently assessed as a treatment for endophthalmitis in rabbits caused by infection with *S. aureus* and MRSA strains. Although bacterial colonies were still present, both strains were more effectively killed by the vancomycin-modified nanoparticles compared to vancomycin alone. Histological analysis also showed that rabbits had lower levels of inflammation when treated with the particles compared to vancomycin treatment alone.

The studies listed above show that antibiotic–nanoparticle systems can selectively kill antibiotic-resistant bacteria for aPDT. However, consideration should be given to the method of antibiotic resistance by a given bacteria, as this strategy is limited by bacteria that have adapted their antibiotic-binding sites. Also, traditional problems associated with antibiotics still exist, including exposure of antibiotics to non-target bacteria in the microbiota, which can damage commensal bacteria and increase the risk of antibiotic resistance developing and spreading among the microbial community [98].

### High-selectivity nanoparticle-based active targeting in aPDT

Recent advances in nanoparticle delivery systems for aPDT have led to the highly selective targeting of bacterial cells using glycans, antibodies, and molecularly imprinted polymers. Here, we will outline the nanoparticle-based systems that have been used, with a summary of the experimental details outlined in Table 1.

#### Glycans

Glycans (sugars) offer a highly selective targeting method by recognising lectins on target cells. For high-affinity binding, lectins rely on multivalent glycan structures. Bacteria use surface lectins to recognise glycan structures on host cell surfaces for attachment and invasion [99]. With the ability of nanoparticles to present multiple glycan structures on their surfaces, these ‘glyconanoparticles’ can mimic the natural glycan presentation found on host cells and create high affinity and selective bacterial binding. The density of glycans on the nanoparticle surface is easily controlled by diluting the glycans with other ‘spacer’ ligands, optimising lectin binding. As glyconanoparticles mimic glycans (‘glycomimetics’) found on the surfaces of human cells, glycans offer biocompatibility and aqueous solubility to drug delivery systems [100, 101].

Zhang et al*.* synthesised silver nanoparticles (*ca.* 160 nm) modified with a galactose-based polymer for targeting *P. aeruginosa* and *S. aureus* [102]. The polymer also contained the photosensitiser 4,4-difluoro-4-bora-3a,4a-diaza-s-indacene or ‘BODIPY’. Bacterial and mouse cell cultures (NIH3T3 cell line) were treated with the galactose- and photosensitiser-modified nanoparticles and imaged. The images showed that the nanoparticles selectively located to the bacterial cells, with no nanoparticles detected in the mouse cell samples. Modified nanoparticles were then added to in vitro bacterial cultures and irradiated, revealing a minimal inhibitory concentration (MIC) of the nanoparticles of 50 pmol/mL. The authors also investigated the bacterial morphology before and after aPDT treatment using SEM. The SEM images showed that bacterial membranes of both *P. aeruginosa* and *S. aureus* were damaged after aPDT treatment in the presence of these nanoparticles. The particles were also found to have good biocompatibility with NIH3T3 cells at 400 pmol/mL.

Zhao et al*.* made use of an antibacterial system that combines PDT and PTT, through near infrared (NIR) laser irradiation [103]. Glycomimetic and pH-responsive gadofullerene-based nanoparticles (171–187 nm) were developed to target drug-resistant *P. aeruginosa* infections. Polymers presenting galactose and fucose residues were used to target the *P. aeruginosa* surface lectins: LecA and LecB, respectively. These lectins are also required by *P. aeruginosa* to form biofilms [104], making them a suitable target for both planktonic and biofilm infections. The glycomimetic polymers self-assembled at physiological pH, encasing the photosensitiser indocyanine green. The nanoparticles were also equipped with a hydrophobic pH-responsive core (2-(diisopropylamino) ethyl methacrylate, DPA) that disassociated upon protonation at pH 6.0 (acidic environments are often associated with infection sites), releasing the photosensitiser at the site of infection. Zhao et al*.* demonstrated that nanoparticles modified with both galactose and fucose eradicated *P. aeruginosa* cultures following 5 min NIR irradiation (808 nm, 2 W/cm^2^) [103]. These nanoparticles were assessed against *P. aeruginosa* biofilm cultures, displaying significant biofilm dispersion (80%) and inhibition (85%) that the authors owe to the ability of the particles to bind both LecA and LecB surface lectins. The nanoparticles had anti-adhesive properties by blocking *P. aeruginosa* from binding to a human lung epithelial cell line, showing good biocompatibility with human cells. These nanoparticles were then assessed for their antibacterial effects in vivo, using mice with *P. aeruginosa*-infected lungs as a model for pneumonia. One hour after the nanoparticles were administered, the chest of the infected mice was irradiated (808 nm, 2 W/cm^2^) for 5 min (with 1 min interval for each irradiation spot). Irradiation of the infected areas was repeated daily for 5 days. The antibacterial effects were compared against buffer and non-irradiated mice, with the nanoparticles significantly reducing the bacterial load (60–80% reduction in colony-forming units) and exhibiting lower levels of inflammation. These findings confirmed the potential of this system for managing drug-resistant *P. aeruginosa* infections, highlighting the strengths that multivalent glycan interactions and multimodal light-based therapies can offer.

Lu et al*.* designed polymeric nanoparticles with different densities of galactose to target either *P. aeruginosa* or *S. aureus* [105]. Nanoparticles had a photosensitiser (BODIPY) core that was co-polymerised with galactose and cationic 2-(dimethylamino)ethyl methacrylate (DMAEMA), generating galactose-modified nanoparticles (16–18 kDa) with different quantities of galactose: 29.3, 45.8 or 62.2%. Nanoparticles with higher quantities of galactose residues exhibited lower cytotoxicity towards NIH3T3 cells. However, the cells were not irradiated in the presence of the nanoparticles. To assess antibacterial activity, all nanoparticles were separately mixed with Gram-negative (*P. aeruginosa*) and Gram-positive (*S. aureus*) bacterial cultures and irradiated. After irradiation, all the nanoparticles exhibited antibacterial activity toward both *P. aeruginosa* and *S. aureus*. Nanoparticles containing 45.8% and 29.3% galactose resulted in reduced viability for both types of bacteria at concentrations as low as 0.3 nmol mL^−1^. Nanoparticles containing 62.2% galactose were more efficient against *S. aureus* at the lowest concentrations tested (0.3 nmol mL^−1^); however, they were more efficient against *P. aeruginosa* at all of the other concentrations investigated. SEM analysis demonstrated that these particles caused cell death through singlet oxygen damage to the bacterial cell wall. While these results show promising selectivity for the pathogens, the authors note that the interaction between the nanoparticles and the bacteria does not appear to be linked to glycan–lectin binding, with imaging experiments suggesting the interaction is predominantly electrostatic.

Using a glucose-modified porphyrin-based polymer (poly-5,15-diphenyl(2,5′-dithienylen)-10,20-di(3,5-di-O-TEG-phenyl), Khan et al*.* synthesised polymeric nanoparticles (poly-5,15-diphenyl(2,5′-dithienylen)-10,20-di(3,5-di-O-TEG-phenyl)) that contained photosensitiser and acetylated glucose to target *B. subtilis* and *E. coli* [106]. The viability of both cultures was reduced by 99% after irradiation and the particles exhibited low dark toxicity, as judged by an 8% reduction in cell viability for cultures that were kept in the dark. These findings demonstrated that the proposed glycan-based system had effective antibacterial activity against both Gram-positive and Gram-negative bacteria.

Staegemann et al*.* developed polymeric particles (hyper branched polyglycerol) that were modified with a porphyrin-derivatised photosensitiser and different densities of mannose (20–110 residues) to target *S. aureus* [107]. Through surface plasmon resonance (SPR) studies, nanoparticles with the higher density of mannose (110 residues) showed the highest binding affinity (140 nM) to the mannose-specific lectin: Concanavalin A. *S. aureus* cultures were mixed with each of the nanoparticles (with different mannose densities) or with photosensitiser alone for 30 min and then irradiated. The nanoparticles with the highest density of mannose (110 residues) completely eradicated *S. aureus* cells at 10 nM, with no detectable dark toxicity. Photosensitiser alone also showed complete eradication of the *S. aureus* cultures in both light (10 and 100 μM photosensitiser concentration) and dark (100 μM photosensitiser concentration) conditions, demonstrating that the mannose residues improved selective aPDT-induced bacterial cell killing. The authors suggested that this selective killing was likely due to the multivalent presentation and binding of the mannose residues to the bacterial cells, enhancing binding affinity. The higher densities of mannose (> 58 residues) were also shown to improve aqueous solubility of the nanoparticles, reducing aggregation [107]. However, the nanoparticle photokilling ability was inhibited in the presence of horse serum. Further investigation showed that this inhibition may be due to serum protein–nanoparticle interactions as photosensitiser quenching was observed with increasing concentrations of BSA, indicating that quenching may be due to proteins present in the environment. While further investigation is required for in vivo therapy application, the findings highlight the importance of multivalent glycan presentation to achieve high-affinity lectin targeting.

Planas et al*.* used cationic mesoporous silica nanoparticles modified with mannose to target *E. coli* and *P. aeruginosa* [108]. Nanoparticles were modified with positively charged amino groups (amino-modified nanoparticles, *ca.* 200 nm) or a 3:1 mixture of amino and mannose residues (mannose-modified nanoparticles, *ca.* 180 nm). The photosensitiser, methylene blue, was then adsorbed onto the modified nanoparticles. The mannose-modified nanoparticles exhibited higher photosensitiser adsorption levels (94%) compared to the amino-modified nanoparticles (73%). Both nanoparticle types and photosensitiser alone were mixed with planktonic cultures of *E. coli* or *P. aeruginosa* for 30 min and irradiated at 652 nm. Mannose-modified nanoparticles were more effective at inducing *P. aeruginosa* cell death (10^8^ CFU/mL reduction at 16 J/cm^2^) than the amino-modified nanoparticles (10^5^ CFU/mL reduction at 16 J/cm^2^). However, while both types of nanoparticles induced a reduction in *E. coli* cell viability (10^7^ CFU/mL reduction), there was no significant difference between the two nanoparticle types and thus, no increased selectivity through mannose binding for the *E. coli*. As optimal glycan binding can be presentation- and density-dependent (see Staegemann et al*.* [107]), the mannose density in the nanoparticles used by Planas et al*.* [108] may not have been optimal for *E. coli* binding and could explain the non-selective binding results observed for *E. coli*.

As shown in the above studies, the extent of pathogen selectivity by glycomimetic nanoparticles can be fine-tuned by altering the glycan structure, density, and presentation. However, as glycans are used widely in nature, simple structures can be promiscuous, limiting their selectivity and requiring optimisation for different pathogens, with binding affinity and selectivity levels dependent on the target lectin.

#### Molecular imprinting

**Fig. 7:**
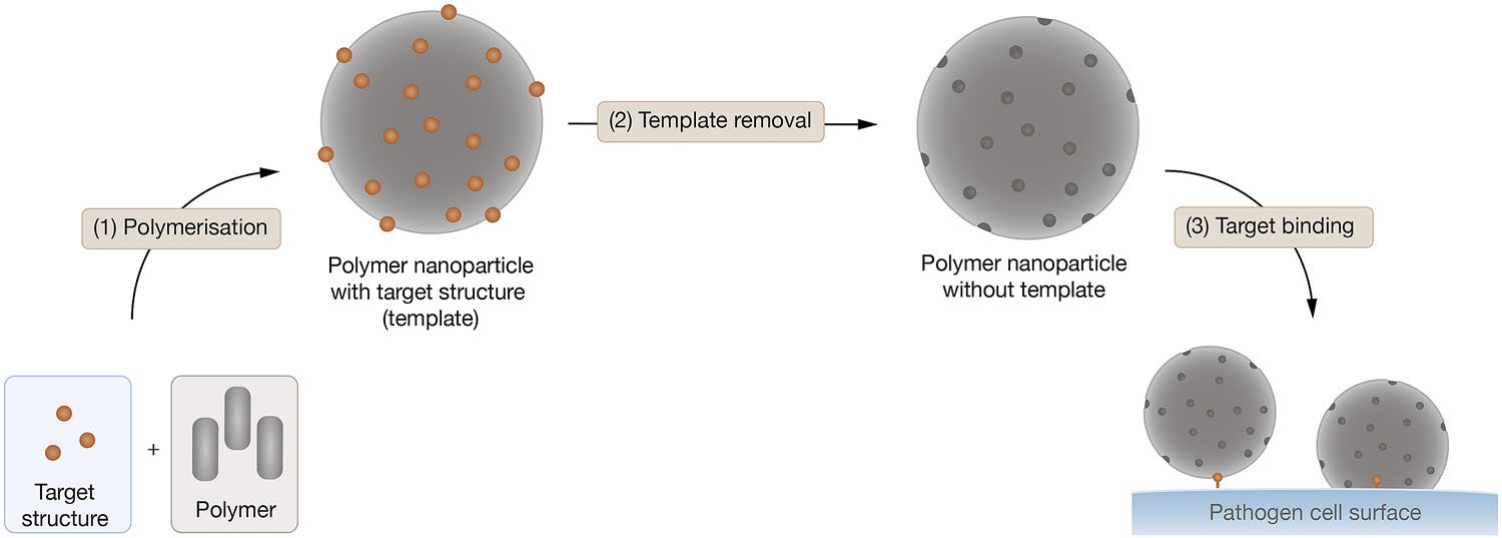
Overview of generation of molecular imprinted nanoparticles for targeting pathogens

Molecular imprinting typically involves mixing target structures with polymers and then removing the target structures from the polymer, leaving an ‘imprint’ (or binding cavity) of the target structure in the polymer [109] (see Fig. 7). This technique offers high-affinity bacterial binding that is highly stable and selective. The approach is generally cost effective as only small quantities of targeting ligands are needed as a template molecule [110].

Long et al*.* designed imprinted polymeric (acrylamide-based) nanoparticles to target lipopolysaccharide found on *P. aeruginosa* [111]. Based on an ‘inverse microemulsion polymerisation’ method, the target structure (lipopolysaccharide) was incubated with the polymeric structures and then removed, forming binding sites that recognise *P. aeruginosa*. A selection of nanoparticles was prepared: lipopolysaccharide-imprinted, lipopolysaccharide-imprinted containing a fluorescent dye, and lipopolysaccharide-imprinted containing the photosensitiser methylene blue. Control nanoparticles lacked lipopolysaccharide imprinting but contained the dye (fluorescent control) or lacked both lipopolysaccharide-imprinting and the dye (non-fluorescent control). Through fluorescence polarity experiments and using a fluorescent lipopolysaccharide probe, Long et al*.* showed that all lipopolysaccharide-imprinted nanoparticles had a strong binding affinity for free lipopolysaccharide (*K*_d_ = 6.6–22.4 nM)[111]. The imprinted nanoparticles were shown to selectively recognise *P. aeruginosa *in vitro (flow cytometry experiments) and in vivo (fluorescence imaging in rabbit keratitis and mouse meningitis models). For in vitro confirmation, binding to either *P. aeruginosa* or *E. coli* was compared between fluorescent lipopolysaccharide-imprinted nanoparticles and fluorescent control nanoparticles. These experiments showed that imprinted nanoparticles selectively recognised *P. aeruginosa*, showing an increased fluorescence signal (3.6–4.9 times) compared to control particles. In the in vivo studies, the lipopolysaccharide-imprinted particles selectively accumulated at the infection site, with significantly elevated fluorescent intensity compared to fluorescent control particles. Binding was due to lipopolysaccharide binding as pre-incubation with free lipopolysaccharide diminished fluorescent intensities in both in vitro and in vivo experiments. Imprinted and control particles containing the photosensitiser methylene blue were used for PDT analysis. *P. aeruginosa* cultures were mixed with either nanoparticles or methylene blue alone and irradiated. A greater reduction in *P. aeruginosa* cell viability was found in the presence of lipopolysaccharide-imprinted nanoparticles that were loaded with methylene blue, with around a 2000-fold reduction compared to the control (no particles or free methylene blue).

Molecular imprinting is a promising targeting strategy for aPDT. However, the technique currently relies on relatively small molecule templates that can be isolated or synthesised. Nonetheless, molecular imprinting offers a highly selective targeting technique for aPDT.

#### Antibodies

The high specificity and affinity of antibody**–**antigen interactions offer great potential for their use as a highly selective targeting agent for aPDT [112]. For example, Wang et al*.* synthesised magnetic iron oxide nanoparticles modified with the photosensitiser hematoporphyrin and a monoclonal anti-MRSA antibody to target *S. aureus* [113]. Planktonic MRSA cultures were mixed with mouse fibroblast cells (L-929) and nanoparticles (500 µg/mL) and irradiated. MRSA cells were completely eradicated in the presence of antibody-modified nanoparticles but no significant toxicity against the L-929 cell line was detected. The selectivity of the particles was further highlighted in the capture ability experiments, where *S. aureus* were mixed with L-929 cells and nanoparticles for 30 min, before being separated from the sample using a magnet. The antibody-modified nanoparticles captured *ca.* 96% of *S. aureus* cells in the mixed cell sample, being much more efficient at capturing bacteria than nanoparticles that lacked antibodies (*ca.* 6%). Through fluorescence imaging (LIVE/DEAD staining), Wang et al*.* also demonstrated that the antibody-modified nanoparticles significantly reduced in vivo MRSA culture viability to 38% in infected wounds (mouse) after 60 min of LED irradiation.

Khlebtsov et al*.* targeted MRSA and methicillin-susceptible *S. aureus* strains developing antibody-modified gold nanoparticles [114]. The nanoparticles were coated with BSA and modified with an anti-*S. aureus* antibody (human anti-*Staphylococcal* immunoglobulin from Microgen, Russia) and the phthalocyanine-derivative photosensitiser Photosens™. The nanoparticles also functioned as imaging agents, emitting red fluorescence that was detectable by fluorescence microscopy or eye (UV**–**Vis illumination). Antibody-modified nanoparticles were mixed with planktonic *S. aureus* cultures and irradiated, where 90% reduction in cell viability was observed for all bacterial strains. However, *S. aureus* cell viability was also reduced under dark conditions and with control nanoparticles that lacked antibody.

Dai et al*.* developed star-shaped nanoparticles for targeted photokilling of a multi-drug resistant *Salmonella* strain (DT104) using an anti-*Salmonella* DT104 antibody (M3038) for selective recognition [115]. Particles had an iron core and gold shell that was modified with methylene blue and anti-*Salmonella* DT104 antibody. The iron core allowed captured bacteria to be separated from the blood by a magnet. The gold shell was used to improve the ease of surface modification, reduce potential toxicity from the iron core, and improve stability during the high-magnetic**–**moment during sensing. As well as PDT effects from methylene blue excitation, the gold shell allowed nanoparticles to generate heat under NIR light for PTT-induced killing. To assess capture efficiency of the nanoparticles, rabbit blood was spiked with *Salmonella* DT104, mixed with the nanoparticles and the nanoparticle-captured bacteria were collected using a bar magnet. The capture efficiency of the nanoparticles for the *Salmonella* strain was 97%. Complete eradication of the *Salmonella* strain was observed after irradiation of the captured bacterial samples. This complete bacterial eradication was due to the combined effects of PDT and PTT after NIR irradiation, as methylene blue alone (PDT effects only) and nanoparticles that lacked methylene blue (PTT effects only) did not eradicate bacteria. These particles are also able to sense *Salmonella* DT104 infection, as shown through fluorescence imaging studies. Dai et al*.* highlighted the multifunction ability that nanoparticles can offer, being able to sense, capture, and eradicate multi-drug resistant bacteria [115].

These antibody-targeting systems demonstrate the high specificity and selectivity that antibody recognition offers. However, antibody production can be a lengthy and expensive process [116]. In addition, for clinical applications, considerations should be given to the potential immunogenicity of antibody constructs [117] and the loss of effective targeting if bacteria mutate their antibody-binding structure [118].

## Conclusions

Despite the large body of research that has shown the viability of aPDT as an alternative strategy for treating some of the most robust infections (drug-resistance and biofilm infections), clinically approved aPDT remains low. Finding ways to overcome the biocompatibility and selectivity limitations of current aPDT strategies is crucial to improving its therapeutic efficacy and reducing its toxicity, so it can reach the clinic. As shown above, nanotechnology-based targeting techniques offer a promising strategy for improving these aPDT outcomes, addressing solubility and target recognition, with ligands, such as glycans, antibodies, and imprinted polymers offering highly selective approaches to target pathogenic bacteria.

## Notes

- Reprinted from Acta Biomaterialia, X. Lu, R. Chen, J. Lv, W. Xu, H. Chen, Z. Ma, S. Huang, S. Li, H. Liu, J. Hu and L. Nie, High- resolution bimodal imaging and potent antibiotic/photodynamic synergistic therapy for osteomyelitis with a bacterial inflammation-specific versatile agent, 99, 363-372, Copyright (2019), with permission from Elsevier.
- Reprinted from Colloids and Surfaces B: Biointerfaces, Z, Zhou, S, Peng, M, Sui, S, Chen, L, Huang, H, Xu and T, Jiang, Multifunctional nanocomplex for surface-enhanced Raman scattering imaging and near-infrared photodynamic antimicrobial therapy of vancomycin-resistant bacteria, 161, 394-402, Copyright (2018), with permission from Elsevier.
